# SARS-CoV-2 Variant-Specific Infectivity and Immune Profiles Are Detectable in a Humanized Lung Mouse Model

**DOI:** 10.3390/v14102272

**Published:** 2022-10-16

**Authors:** Yunyun Di, Jocelyne Lew, Una Goncin, Anna Radomska, Saurav S. Rout, Bridget E. T. Gray, Steven Machtaler, Darryl Falzarano, Kerry J. Lavender

**Affiliations:** 1 Department of Biochemistry, Microbiology and Immunology, University of Saskatchewan, Saskatoon, SK S7N 5E5, Canada; 2 Vaccine and Infectious Disease Organization, University of Saskatchewan, Saskatoon, SK S7N 5E3, Canada; 3 Department of Medical Imaging, University of Saskatchewan, Saskatoon, SK S7N 0W8, Canada; 4 Department of Pathology and Laboratory Medicine, University of Saskatchewan, Saskatoon, SK S7N 5E5, Canada; 5 Animal Care and Research Support, Research Excellence and Innovation, University of Saskatchewan, Saskatoon, SK S7N 5E5, Canada; 6 Department of Veterinary Microbiology, University of Saskatchewan, Saskatoon, SK S7N 5B4, Canada

**Keywords:** SARS-CoV-2, humanized mice, variants of concern, immunity

## Abstract

Small animal models that accurately model pathogenesis of SARS-CoV-2 variants are required for ongoing research efforts. We modified our human immune system mouse model to support replication of SARS-CoV-2 by implantation of human lung tissue into the mice to create TKO-BLT-Lung (L) mice and compared infection with two different variants in a humanized lung model. Infection of TKO-BLT-L mice with SARS-CoV-2 recapitulated the higher infectivity of the B.1.1.7 variant with more animals becoming infected and higher sustained viral loads compared to mice challenged with an early B lineage (614D) virus. Viral lesions were observed in lung organoids but no differences were detected between the viral variants as expected. Partially overlapping but distinct immune profiles were also observed between the variants with a greater Th1 profile in VIDO-01 and greater Th2 profile in B.1.1.7 infection. Overall, the TKO-BLT-L mouse supported SARS-CoV-2 infection, recapitulated key known similarities and differences in infectivity and pathogenesis as well as revealing previously unreported differences in immune responses between the two viral variants. Thus, the TKO-BLT-L model may serve as a useful animal model to study the immunopathobiology of newly emerging variants in the context of genuine human lung tissue and immune cells.

## 1. Introduction

Two years after the first reported COVID-19 case, SARS-CoV-2 continues to challenge healthcare, educational and economic systems worldwide despite the extensive rollout of highly effective vaccines worldwide. SARS-CoV-2 continues to circulate widely in many areas with major variants of concern (VOC) such as the Alpha (B.1.1.7), Delta (B.1.617.2) and recently Omicron (B.1.1.529) variants initiating sequential and significant expansions in caseloads worldwide. The most significant variants to date have developed mutations that result in greater infectivity, altered pathogenicity, decreased vaccine efficacy or a combination of these factors [1].

Numerous notable mutations have emerged in VOC, including within the spike protein that is used by the virus to attach to and enter target cells [2]. Spike mutations identified during the early stages of the global pandemic including D614G, N501Y, N439K and Y453F, were shown to increase infectivity via enhanced binding to ACE2 and for certain mutations resulted in reduced neutralizing activity of monoclonal antibodies (mAb) [3,4,5,6,7]. Deep mutational scanning further identified E484K and S477G spike mutations as ranking prominently in reducing neutralization or escape of the mAbs tested [2,8]. Mutations L452R and T478K in the Delta variant are associated with reduced viral neutralization by vaccine-induced antibodies [9] and clinical data suggested that spike mutations were able to reduce vaccine-mediated protection from infection [10,11,12,13]. In addition, non-spike mutations in viral proteins such as N, orf6, and orf9b might also play a role in differential evasion of innate immunity by VOCs and altered pathogenesis [14].

With some countries experiencing low vaccine uptake, short-lived humoral immunity to current VOC and inequitable vaccine access worldwide, the evolution and emergence of additional SARS-CoV-2 VOC is likely to continue. New VOC are likely to emerge that possess mutations that can impact viral infectivity, pathogenicity, vaccine efficacy and susceptibility to therapeutics. Thus, animal models that can be used to rapidly screen newly emerging variants and specific mutations for their impact on these parameters are crucial to continued success in the battle against SARS-CoV-2. Numerous animal models such as non-human primates, ferrets and hamsters have been employed in COVID-19 research [15,16], each with their own set of advantages and caveats [17,18]. In all animal models, species-specific differences in the sequence and structure of ACE2 [19,20], cellular physiology and immune responses [21,22] may impact the ability to accurately assess differences in VOC pathogenicity and infectivity as well as to test therapeutics with species-specific modalities such as interferons [23], and therapeutic antibodies.

Although mice are often the preferred animal model due to their small size and ease of acquisition and use, they are not natural hosts for all SARS-CoV-2 variants and often require viral adaptation or transgenic expression of human ACE2 [24,25]. In transgenic mouse models, the ectopic expression of ACE2 may not fully recapitulate systemic expression levels in humans complicating interpretation of infectivity studies and the requirement for viral adaptation precludes the evaluation of VOCs in their natural form. Additionally, COVID-19 is immune driven and differences in human and animal immune components and function could confound interpretation of immunopathogenesis studies in animal models. For example, NKG2D, an activating receptor on NK cells, is elevated in severe COVID-19 patients [26] suggesting the receptor has an important role in disease. However, the ligands for NKG2D are different in humans versus mice which may affect its biological function [27]. Thus, such differences may increase the difficulty of extrapolating the experimental outcomes observed in mouse models to humans.

Researchers have previously shown that reconstitution of immunocompromised mice with a human immune system (HIS mice) facilitates the study of viral infections and evaluation of therapeutics against viruses with hematopoietic tropism that do not normally replicate in mice, such as HIV-1 [28]. A variety of HIS models have been developed through the implantation of human immune cells and/or tissues into a number of immunocompromised background mouse strains [29]. We developed a bone marrow, liver and thymus (BLT) mouse model using C57BL/6 *Rag^−/−^γ_c_^−/−^CD47^−/−^* (TKO) mice [30,31]. TKO-BLT mice have a highly functional human immune system and have proven useful for HIV-1 research [32,33]. However, HIS models do not support human-specific viral infections that do not have significant hematopoietic tropism such as respiratory viruses that preferentially infect cells within the human lung. To overcome this, HIS mice can be additionally implanted with human lung tissue that is autologous to the reconstituted human immune system to support a range of respiratory viruses including coronaviruses [34,35,36,37]. In this study, we modified our TKO-BLT model to support SARS-CoV-2 infection through the subcutaneous implantation of autologous human lung tissue to produce the TKO-BLT-Lung mouse (TKO-BLT-L). The TKO-BLT-L mouse developed well vascularized human lung organoids populated with human immune cells, supported SARS-CoV-2 infection, recapitulated the differential levels of infectivity seen in humans and revealed previously unreported distinct immune profiles when challenged with an early B lineage (614D) SARS-CoV-2 virus compared to the B.1.1.7 VOC. Thus, the TKO-BLT-L model appears useful for the evaluation of differences in viral infectivity and immunopathobiology of SARS-CoV-2 VOCs within the context of genuine human lung tissue and a bona fide human immune response.

## 2. Materials and Methods

### 2.1. Ethics Statement

Human fetal thymus, liver and lung tissues for reconstitution of humanized mice were obtained through anonymous donations with informed written consent via Advanced Bioscience Resources (Alameda, CA, USA) under the University of Saskatchewan Research Ethics Board Bio ID-371. All animal studies were performed under University of Saskatchewan’s Animal Research Ethics Board protocols 20180079 and 20200016 and adhered to Canadian Council on Animal Care guidelines.

### 2.2. Humanized TKO-BLT-L Mice

TKO-BLT mice were generated as previously described [31,32] with the additional implantation of autologous human lung tissue to make TKO-BLT-Lung (L) mice. Briefly, a 1 mm^3^ piece of 17–22-week gestation human fetal thymus and liver was placed under the kidney capsule of 6–10-week-old male and female TKO mice followed by injection of 1-2 × 10^6^ autologous liver-derived CD34^+^ hematopoietic stem cells. Two pieces (~2–4 mm^3^) of autologous fetal lung tissue were then implanted subcutaneously into the back (right and left back or upper and lower back). Subcutaneous wounds were closed with surgical glue.

### 2.3. Analysis of Human Immune Cell Reconstitution

TKO-BLT-L mice were bled at 8- and 12-weeks post-surgery. Blood leukocytes were purified using RBC Lysis buffer (BioLegend, San Diego, CA, USA). Lung organoids were collected and digested using 60 mg/mL collagenase D (Roche, Basil, Switzerland) and 10 U/mL DNAse I for 1 h before passage through 70 mm filters. Leukocytes were then isolated using a 40%/70% Percoll gradient. Cells were incubated with anti-human Fc blocker (Thermo Fisher Scientific, Waltham, MA, USA) before antibody staining. Anti-mouse specific CD45, was used to exclude mouse leukocytes. Antibodies used included: CD3 V450, CD4 APC, CD8 APC-eFluor 780, CD14 PC7, CD16 FITC, CD19 PerCP-Cy5.5, CD45 V500, Live/Dead-PE-Texas Red, CD123 PE (eBioscience, San Diego, CA, USA), CD33 PE (Miltenyi, Bergisch Gladbach, Germany), CD56-AF700, CD11c-APC, HLA-DR and Lin-1 (CD3, CD14, CD16, CD19, CD20, CD56)-FITC (Biolegend). Data were acquired on a BD CytoFlex (Beckman Coulter, Brea, CA, USA) and analyzed with FlowJo Version 10.6 (BD Biosciences, Franklin Lakes, NJ, USA).

### 2.4. Ultrasound

Hair was removed from lung implants using depilatory cream 1 day before ultrasound imaging. Mice (*n*  =  5 mice, 6 human lung implants, 15–25 weeks post-transplant) were anesthetized with vaporized isoflurane (maintained at 1.5%) and placed on a heated stage (37 °C) during imaging. Warmed ultrasound gel was applied on the skin over the lung organoid. Organoids were imaged using a preclinical small animal ultrasound system (Vevo3100, FujiFilm VisualSonics, Toronto, ON, Canada) with a high resolution 20 MHz transducer (MX250) fixed to a stepping motor to enable 3D scanning. Both B-mode and non-linear contrast mode data were collected. Poly dispersed lipid-shelled, perfluorobutane-filled microbubbles were synthesized as described in [38]. Contrast enhanced ultrasound imaging in mice was performed after an intravenous bolus injection of 5 × 10^7^ lipid-shelled, perfluorobutane-filled microbubbles in 100 uL saline over 10 s through the tail vein. Lung volumes were obtained and analyzed using Vevo LAB software (FujiFilm VisualSonics).

### 2.5. Immunohistochemical Analysis

Lung organoids were harvested and fixed in 4% paraformaldehyde. Slides were prepared and stained by Prairie Diagnostic Services (PDS) Inc. (Saskatoon, SK, Canada) Tissue sections were stained with Hematoxylin-eosin (H&E) and Alcian-blue periodic acid-Schiff stain to identify microanatomy and mucin secretions, respectively. In addition, lung organoid sections were stained for human epithelial cells (Cytokeratin 19, 1:50, Abcam, Cambridge, UK), endothelial cells (CD34, 1:50, Agilent Technologies, Santa Clara, CA, USA), mesenchymal cells (Vimentin, 1:120K, Sigma-Aldrich, St. Louis, MO, USA), tubulin (1:100, Sigma-Aldrich), club cells (CC10, 1:4000, Santa Cruz Biotech, Dallas, TX, USA), human hematopoietic cells (CD45, 1:50, Agilent Technologies), T cells (CD3, 1:25, Thermo Fisher Scientific), macrophages (CD68, 1:50, Agilent Technologies), B cells (CD20, 1:50, Biocare Medical, Pacheco, CA, USA), plasmacytoid DC (BDCA-2, 1:400, R&D, Minneapolis, MN, USA), ACE2 (1:200, Sigma-Aldrich), TMPRSS2 (1:100, Sigma-Aldrich), and SARS-CoV nucleoprotein (1:6000, Sino Biological, Beijing, China). Following deparaffinization and blocking of endogenous peroxidase, epitope retrieval was performed in a Tris/EDTA buffer (pH = 9) for 20 min at 97 °C. Primary antibodies were applied for 30 min at room temperature, and binding of the primary antibodies was detected using an HRP-labelled polymer detection reagent (Agilent Technologies). The staining was visualized using 3,3′-diaminobenzidine tetrahydrochloride (DAB) as the chromogen (Agilent Technologies). The IHC slides were scanned using Aperio Virtual Microscopy System (Leica Biosystems, Wetzlar, Germany) at 20X resolution and viewed using Aperio ImageScope software (version 12.4.3). Organoids stained with H&E were each assessed over 12 observed lesion parameters (vascular dilation, erythrocyte extravasation, vascular/perivascular lymphocyte infiltration, epithelial wall lymphocytic infiltration, proteinaceous exudate in the lumen, desquamated epithelial cells, macrophages or lymphocytes in the lumen, apoptotic bodies, granulocytes, type II pneumocyte hyperplasia, thrombus formation, lymphocyte follicle formation) and assigned scores from 0 (not present) to 3 (strong presence). The sum of the 12 scores served as the pathology index for each sample. Staining indices were collated from three independent pathologists and histological comparisons were assessed in a masked fashion.

### 2.6. Viruses

The early B lineage (614D) SARS-CoV-2 virus: hCoV-19/Canada/ON/VIDO-01/2020, (GISAID: EPI_ISL_425177) was isolated from a clinical specimen obtained at the Sunnybrook Research Institute/University of Toronto (Toronto, ON, Canada) on Vero’76 cells (ATCC, Manassas, VA, USA). The B.1.1.7 (Alpha) variant was isolated on Vero’76 cells from a clinical specimen kindly provided by Graham Tipples and Kanti Pabbaraju at Alberta Health Services (Edmonton, AB, Canada). Virus was initially passaged at a 1:1000 dilution on mycoplasma-free Vero’76 cells (ATCC) in Dulbecco’s Modified Eagle’s Medium (Sigma) containing 1% L-glutamine and 1μg/mL of TPCK-trypsin and harvested when 80% cytopathic effect (CPE) was evident. The virus stock was clarified by centrifugation at 4816× *g* for 10 min and stored at −80 °C until thawed for infections. The virus stock was titrated on Vero’76 cells by conventional TCID_50_ assay.

### 2.7. Viral Challenge

TKO-BLT-L mice with sufficient human immune reconstitution (2 × 10^5^ human CD45^+^ cells/mL blood) and lung organoid 1–1.5 cm in diameter were included in experiments. SARS-CoV-2 (1 × 10^5^ TCID in 50 μL saline) was directly injected into each human lung organoid. Mice were grouped to control as closely as possible for lung organoid size, mouse and human donor sex and level of human immune reconstitution. Lung organoids were collected on day 2, 5, 7 and 9 post-challenge. Lung organoids were sectioned into thirds and each section used for infectious virus assay, real time RT-PCR and immunohistochemistry, respectively.

### 2.8. Quantification of Infection

Lung sections were weighed and placed in DMEM before being homogenized in a Tissuelyser II Homogenizer (Qiagen, Hilden, Germany) at 30 Hz for 6 min. For live virus assays, tissue homogenates were clarified by centrifugation at 5000× *g* for 5 min and then serially diluted 10-fold in DMEM supplemented with 2% heat-inactivated FBS, 1x L-glutamine, and 1x penicillin–streptomycin. Sample volumes of 50μL were added to 96-well plates of 95% confluent Vero’76 cells in triplicate and incubated for 5 days at 37 °C with 5% CO_2_ before scoring for the presence of cytopathic effects. For real time RT-qPCR quantification, viral RNA from 30 mg of tissue was extracted using a RNeasy Plus Mini kit (Qiagen, Hilden, Germany). The SARS-CoV-2 E-specific real time RT–qPCR assay was used for the detection of viral RNA. RNA was reverse transcribed and amplified using the primers E_Sarbeco_F1, E_Sarbeco_R2, and probe E_Sarbeco_P1 using the QuantiFast Probe RT-PCR kit (Qiagen). Quantification of viral genome copy numbers was done using a standard curve generated in parallel for each plate using synthesized DNA. The StepOne Software (Thermo Fisher Scientific) was used to calculate the cycle threshold values.

### 2.9. Human Cytokine and Chemokine Profile

The human cytokine and chemokine profile was determined using RT2 Profiler PCR Array Formats E384 (4 × 96) option (Qiagen) according to manufacturer’s instructions. The RNA was reverse transcribed into cDNA using a RT^2^ First Strand kit (Qiagen). The cDNA was then added to RT^2^ SYBR Green ROX qPCR Mastermix (Qiagen) and 10 μL aliquots used across the RT^2^ Profiler^TM^ PCR Array. Cycle conditions were: 10 min at 95 °C; 40 cycles of 15 s at 95 °C, and 60 s at 60 °C using the QuantStudio™ 6 Pro Real-Time PCR System (Thermo Fisher Scientific). The Cq values was determined using Design and Analysis version 2.6. Data were then analyzed using web-based RT² Profiler PCR Data Analysis software (Qiagen GeneGlobe online tool: https://dataanalysis2.qiagen.com/pcr (accessed on 1 January 2022). Gene expression levels were quantified relative to the average arithmetic mean values obtained for housekeeping genes ACTB, B2M, GAPDH, HPRT1, and RPLP0.

### 2.10. Statistical Analysis

Statistical calculations were performed with Prism 9.0 (GraphPad Software, La Jolla, CA, USA). For comparisons of two separate parametric datasets, unpaired two-tailed Student’s t-tests were performed and one-way ANOVA with Tukey’s post-test used for multiple comparisons. For all statistical comparisons *p* < 0.05 was considered significant.

## 3. Results

### 3.1. Subcutaneously Implanted Human Lung Tissue Expands into Highly Vascularized Lung Organoids

**Figure 1:**
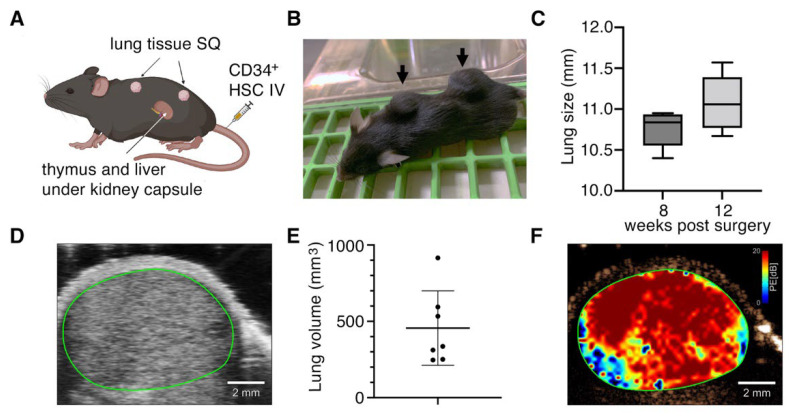
Implanted human lung tissue develops into well vascularized subcutaneous lung organoids on TKO-BLT mice. (**A**) Schematic of humanization procedure to produce TKO-BLT-L mice. Created with BioRender.com. (**B**) Representative mouse with subcutaneous lung organoids established on the upper and lower back (arrows). (**C**) Average lung diameter at 8- and 12- weeks post-surgery (wps) ±SD (n = 88, *p* = 0.13). (**D**) Representative B-mode ultrasound imaging of subcutaneously implanted lung organoid (green line) (n = 5). (**E**) 3D-scans were used to determined implant volumes with an average of 455.7 ± 243.8 mm^3^ (n = 7) and (**F**) Representative perfusion map generated from implanted lung organoids (n = 5). Data in D-F collected at 15–24 wps.

We previously developed triple knock-out C57BL/6 *Rag*^-/-^*γ_c_*^-/-^*CD47*^-/-^ mice reconstituted with a human immune system using the bone marrow, liver, thymus method (TKO-BLT mice) [30,31] for use in studies with HIV-1 [32,33,39,40] and other viral infections [41] that have tropism for human hematopoietic cells. Since SARS-CoV-2 infects human but not mouse lung tissue [42], we modified our TKO-BLT model by subcutaneously implanting two pieces of ~2–4 mm^3^ autologous lung tissue in addition to the BLT humanization procedure to produce TKO-BLT-Lung (L) mice (Figure 1A). Implanted lung tissue expanded and became visible as two distinct subcutaneous lung organoids on the backs of the mice (Figure 1B). Lung organoid diameter was measured at 8- and 12- weeks post-surgery (wps) using a circle ruler and palpation. The average lung diameter at 8 wps was 10.7 mm (SD ± 0.26) and 11.2 mm (SD ± 0.45) by 12 wps (Figure 1C). B-mode imaging revealed the location of a distinct lung organoid between the skin and muscle layer (Figure 1D). The volume of lung organoids was determined using 3D ultrasound scanning, and averaged 456 mm^3^ (SD ± 243 mm^3^, n = 7) at 12 wps (Figure 1E). Acoustic angiography was performed to assess the vascularity of implanted organoids and perfusion mapping showed a significant density of blood flow throughout the lung implants (Figure 1F). Overall, subcutaneously implanted human lung tissue, autologous to simultaneously transplanted human immune cells and tissues, developed to a sufficient size for use in experiments and became well vascularized.

### 3.2. Systemic Human Immune Cell Reconstitution Is not Altered by Implantation of Lung Organoids

Peripheral blood was collected at 12- weeks post-surgery to determine if implantation of human lung tissue subcutaneously onto TKO-BLT mice impacted their systemic human immune cell reconstitution. TKO-BLT-L mice had 3.25 × 10^5^ (±6.3 × 10^4^) human CD45^+^ leukocytes/mL of blood at 8 weeks post-surgery (wps) and this increased to 4.69 x 10^5^ (±5.23 × 10^4^) cells/mL 12 wps (Table 1). The frequency of total human CD45^+^ leukocytes, CD4^+^, CD8^+^ and total CD3^+^ T cells, CD19^+^ B cells, total CD33^+^ myeloid cells, CD14^+^ monocytes, CD3^-^CD56^+^ natural killer (NK) cells and both myeloid (lin^-^DR^hi^CD11c^+^) and plasmacytoid (lin^-^DR^hi^CD123^+^) dendritic cells (DC) in the blood was determined by flow cytometry (Table 1). The human immune cell profile in the peripheral blood of TKO-BLT-L mice was comparable with our original TKO-BLT mouse model (Table 1) with no statistically significant changes (*p* ≥ 0.05, 2-tailed *t*-test) observed for any cell type. These data suggested that implantation of human lung tissue subcutaneously onto the backs of mice in addition to the BLT humanization procedure had no effect on the level of systemic human immune reconstitution of the resultant TKO-BLT-L mice.

### 3.3. Lung Organoids Develop Structures and Contain Cell Types and SARS-CoV-2 Entry Molecules Similar to Human Lungs

**Figure 2:**
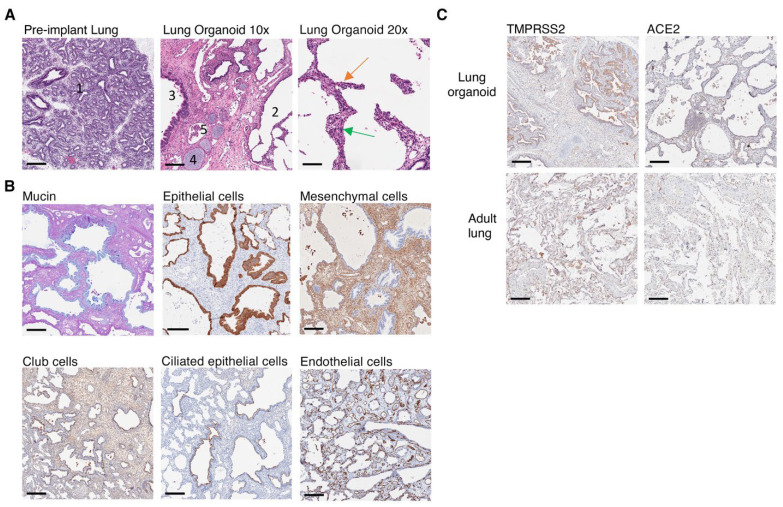
Human lung cells, lung structures and SARS-CoV-2 entry molecules are present in implanted lung organoids. (**A**) Pre-implant lung at glandular/canalicular stage (1). Ectopic lung implant with alveolar structures (2) and bronchial and bronchiolar structures lined by glandular ciliated respiratory epithelium (3), cartilage present in the wall of bronchi (4), and associated blood vessels (5) at ×10 magnification (scale bars, 200 µm). Alveolar structures in lung implant lined by type-1 (green arrow) and type-2 pneumocytes (orange arrow) visualized at ×20 magnification (scale bars, 100 µm). (**B**) Immunohistochemical staining of lung implants showing the presence of human glandular epithelium including human goblet cells (blue mucin, AB/PAS), epithelial cells (cytokeratin 19), mesenchymal cells (vimentin), club cells (CC10), ciliated glandular epithelium (tubulin) and endothelial cells (CD34). (**C**) Expression of ACE2 and TMRPSS2 in implanted lung organoid compared to a healthy lung section from a 71-year-old adult male. (**B**,**C**) Positive staining = brown unless otherwise indicated, ×10 magnification (scale bars, 200 µm). Images represent n = 4 at 15 wps.

To evaluate whether the subcutaneous lung organoids developed key characteristics of human lung tissues we performed immunohistochemical analysis of the organoid structure and its main constituent cell types. Hematoxylin and eosin (H&E) stains of lung organoids showed that the implanted lung organoid developed alveolar structures from pre-alveolar sacs observed in the lung tissue prior to transplant even though the lung was ectopically implanted and not ventilated (Figure 2A). Furthermore, we observed the presence of ciliated epithelium in the bronchus, hyaline airway cartilage, associated seromucinous glands in mucosa and blood vessels (Figure 2A). Type-1 and type-2 pneumocytes were also observed (Figure 2A). AB/PAS staining detected mucus secretions in the lung organoid, suggesting the presence of functional goblet cells (Figure 2B). In addition, specific immunostaining detected the presence of multiple non-hematopoietic human cell types associated with lung tissue including epithelial cells, mesenchymal cells, club cells, ciliated epithelial cells and endothelial cells (Figure 2B). Endothelial cells (CD34) lined interstitial vessels in the implanted lung parenchyma.

SARS-CoV-2 infects human epithelial cells by directly binding to the cell surface angiotensin-converting enzyme 2 (ACE2) receptor, followed by S1/S2 cleavage by transmembrane protease serine 2 (TMPRSS2), resulting in viral entry and replication [43]. Thus, we assessed whether the expression of ACE2 and TMPRSS2 on lung organoids developed from implanted sections of fetal lung tissue would express sufficient quantities of these molecules to support SARS-CoV-2 infection. We detected both ACE2 and TMPRSS2 expression in lung organoids (n = 4) with similar localization and expression levels compared to a healthy human lung section procured from a 71-year-old adult male undergoing biopsy (Figure 2C). These data revealed that the implanted lung organoids contained cell types and developed structures that would normally be expected in a fully developed human lung and expressed the major entry molecules required for SARS-CoV-2 infection.

### 3.4. Implanted Lung Tissues in TKO-BLT-L Mice Become Reconstituted with Human Immune Cells

**Figure 3:**
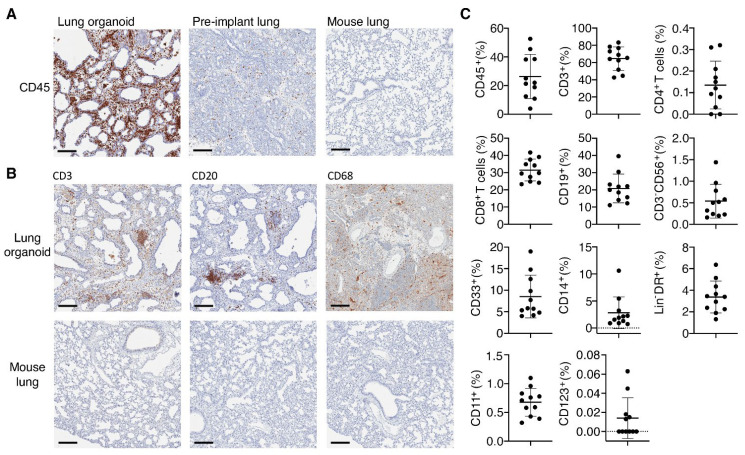
Presence of human immune cells in implanted lung organoids. (**A**) Representative immunohistochemical (IHC) staining for CD45^+^ human immune cells in lung organoids implanted on human immune reconstituted mice compared to pre-implant lung tissue. A mouse lung from a non-humanized TKO mouse served as a control for human specificity of the antibody. (**B**) Representative IHC stains for the presence of human T cells (CD3), B cells (CD20) and macrophages (CD68) in lung organoids on TKO-BLT-L mice. Mouse lung tissue serves as a negative staining control. 15 wps (n = 2), positive staining (brown), ×10 magnification, scale bars, 200 µm. (**C**) Flow cytometric analysis of immune cells isolated from lung organoids between 20- and 25-weeks post-surgery. Frequencies were determined for total human leukocytes (hCD45^+^) cells, myeloid cells (CD33^+^), B cells (CD19^+^), CD4^+^ and CD8^+^ as well as total T cells (CD3^+^), monocytes (CD14^+^), NK cells (CD3^-^CD56^+^), total (Lin^-^DR^hi^) DC and myeloid (CD11c^+^) and plasmacytoid (CD123^+^) DC subsets. Each dot represents an individual lung implant (n =  11 implants). Horizontal lines represent mean  ±  SD. Each cell type is expressed as the frequency of human leukocytes except for CD45 cells, which was calculated from the live cell gate and CD4^+^ and CD8^+^ cells, which are expressed as frequency of CD3^+^ T cells.

Since immune pathology is a major feature of SARS-CoV-2 infection we next assessed whether the human immune cells that systemically reconstitute TKO mice upon BLT humanization would also reconstitute the human lung organoids in TKO-BLT-L mice. Immunohistochemical staining confirmed the presence of human CD45^+^ hematopoietic cells in the human lung organoids, that were also detected at much lower levels in pre-implant lung tissue but not in control mouse lung tissue (Figure 3A). Major immune cell lineages such as T cells (CD3), B cells (CD20) and macrophages (CD68) were all detectable by IHC in the subcutaneous lung organoids (Figure 3B).

To more extensively assess the human immune cell complement in the lung organoids we performed flow cytometric analysis on leukocytes isolated from the lung organoids of 20–25 wps TKO-BLT-L mice produced from two different human donors (Figure 3C). The lung organoids contained the same immune cell types found in the peripheral blood of TKO-BLT-L mice. Frequencies were determined in lung organoids for total CD45^+^ human leukocytes (26.31 ± 15.42), CD33^+^ myeloid cells (8.53 ± 4.96), CD19^+^ B cells (20.80 ± 8.37), total CD3^+^ T cells (64.69 ± 13.53) and each subset within including CD4^+^ (0.14 ± 0.11) and CD8^+^ (31.50 ± 6.39) T cells, CD14^+^ monocytes (2.82 ± 2.91), CD3^-^CD56^+^ NK cells (0.54 ± 0.39), total Lin^-^DR^hi^ DC (3.38 ± 1.48) and each subset within including CD11c^+^ myeloid (21.83 ± 6.94) and CD123^+^ plasmacytoid (0.29 ± 0.38) DC (Figure 3C). The immune cell subsets in a 59-year-old female adult human lung sample were also evaluated as a reference (Table 2). Interestingly, similarly low frequencies of hCD45 (11%) and CD4^+^ T cells (0.46%) were observed in the adult human lung as in the TKO-BLT-L lung organoids. Similar to what is observed with the peripheral blood reconstitution of TKO-BLT mice [32], the frequencies of T and B cells in human lung organoids were relatively comparable to the ex vivo human sample whereas myeloid and NK cell lineages were present but underrepresented (Figure 3C).

### 3.5. Differences in the Viral Infectivity and Persistence of SARS-CoV-2 Variants Were Detectable in TKO-BLT-L Mice

**Figure 4:**
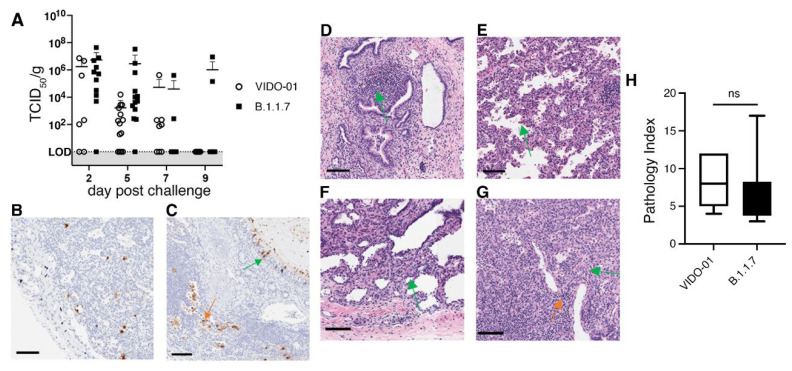
Differences in infectivity and viral persistence between SARS-CoV-2 variants was detectable in TKO-BLT-L mice. TKO-BLT-L mice were challenged via intra-organoid injection of 1 × 10^5^ TCID_50_ of either VIDO-01, an early B lineage (D614) virus, or the B.1.1.7 Alpha variant of concern and (**A**) organoids harvested at days 2, 5, 7 and 9 post-challenge to evaluate the live viral titer per gram of tissue. Each symbol represents an individual lung organoid harvested from TKO-BLT-L mice produced from a total of eight different human donors. Horizontal lines denote mean ±SD. LOD = Limit of detection. Organoids harvested at day 5 post-challenge were either (**B**,**C**) stained for SARS-CoV-2 N protein (brown) or (D-G) H&E stained to assess for SARS-CoV-2-related pathology. N protein staining of (**B**) alveolar macrophages (**C**) ciliated bronchial epithelia (green arrow) and desquamated epithelial cells (orange arrow), (**D**) epithelial cell/mucosa lymphocyte infiltration, (**E**) extravasated erythrocytes, (**F**) type-2 pneumocyte hyperplasia, (**G**) perivascular lymphocyte infiltration (orange arrow) and apoptotic bodies (green arrow). ×20 magnification, scale bars, 100 µm. Representative staining is shown in organoids infected with either VIDO-01 or B.1.1.7. (**H**) Histological scoring of lesions (pathology index) of lung organoids at day 5 infected with either VIDO-01 or B.1.1.5. Unpaired t-test, ns = not significant.

The TKO-BLT-L mice were challenged with 1 × 10^5^ TCID_50_ of SARS-CoV-2 virus by direct injection into lung organoids. Implants were then collected on days 2, 5, 7 and 9 to determine the level of live viral infection in organoids at each time point. Only 68% of lung organoids challenged with early B lineage (614D) VIDO-01 virus had detectable live virus at days 2 and 5 compared to 92% of organoids challenged with the B.1.1.7 Alpha variant (Figure 4A). At 2 days post-challenge the mean viral titer of B.1.1.7 in lung organoids was 5.5 × 10^6^ TCID_50_/g (±1.3 × 10^6^) compared to 1.8 × 10^6^ TCID_50_/g (±3 × 10^6^) in organoids recovered from mice challenged with VIDO-01. The viral titers dropped dramatically to 1.7 × 10^3^ TCID_50_/g (±4.1 × 10^3^) at day 5 in VIDO-01 infected lung organoids while a mean of 2.9 × 10^6^ TCID_50_/g (±9.3 × 10^6^) was recovered from B.1.1.7 challenged mice. Two B.1.1.7 challenged lung organoids still had a detectable live viral titer at day 9 post-challenge but mice challenged with VIDO-01 had all cleared the virus at this time point (Figure 4A). Throughout the duration of infection there were no overt signs of disease (such as weight loss or fever) in the mice (*dns*).

Immunohistochemical staining for the SARS-CoV-2 N protein in lung organoids at day 5 revealed a similar and characteristic distribution of the virus in both viral infections and N protein was detected in alveolar macrophages (Figure 4B) and ciliated epithelial cells (Figure 4C). Similarly, H&E staining detected pathological changes in the infected lung organoids typical of early viral infection including desquamated epithelial cells, vascular, perivascular and epithelial cell lymphocytic infiltration, as well as infiltration in mucosa of non-cartilaginous bronchiolar airways, extravasated erythrocytes in alveolar spaces and lung parenchyma, apoptotic bodies and focal type-2 pneumocyte hyperplasia (Figure 4C–G). However, no hyaline-membrane formation typical in respiratory distress syndrome was observed. Histological scoring showed no significant differences in the extent of lesions in lung organoids infected with VIDO-01 versus the B.1.1.7 virus (Figure 4H). Thus, SARS-CoV-2 infection of the TKO-BLT-L mouse recapitulated features of genuine human SARS-CoV-2 infection including the increased infectivity and persistence of the B.1.1.7 VOC with no observable differences in lung pathology and disease severity compared to an early B lineage (614D) virus.

### 3.6. The Immune Responses to SARS-CoV-2 Infection Differed between Viral Variants and Required the Systemic Human Immune System of TKO-BLT-L Mice to Mediate Viral Clearance

**Figure 5:**
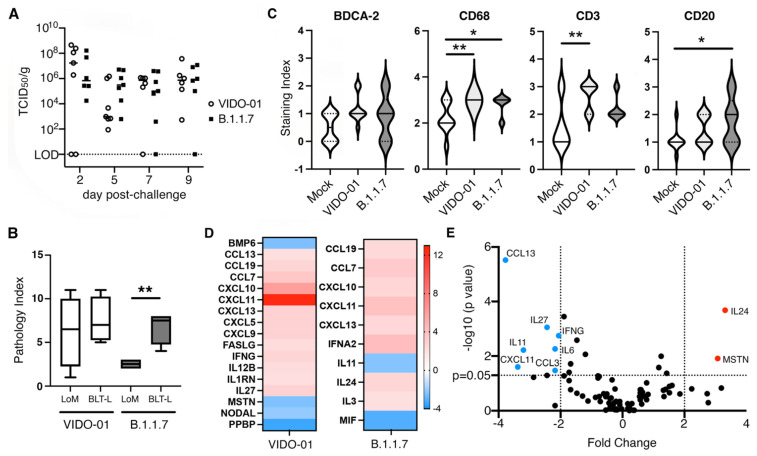
Human immune responses to SARS-CoV-2 in lung organoids. (**A**) Lung organoids implanted onto TKO mice in the absence of a systemic human immune system were challenged with 1 × 10^5^ TCID_50_ of B.1.1.7 or VIDO-01 and infectious viral titers determined at days 2, 5, 7 and 9. Each dot represents an individual lung organoid. Horizontal lines denote mean. LOD = Limit of detection. (**B**) Histological scores of lesions (pathology index) in lung organoids from animals that either were (BLT-L) or were not (LoM) implanted with a human immune system and infected with either VIDO-01 or B.1.1.5 for 5 days. Unpaired t-test. ** = *p* < 0.01. (**C**) Level of human BDCA-2, CD68, CD3 and CD20-specific immunohistochemical staining in lung organoids from uninfected and SARS-CoV-2 variant infected TKO-BLT-L mice at day 5 post infection. * = *p* < 0.05, ** = *p* < 0.01. Solid line denotes mean and dotted lines SD, n = 3 per group. One-way ANOVA with Tukey’s posttest. (**D**) Lung organoids from TKO-BLT-L mice infected with VIDO-01 or B.1.1.7 were assessed for expression of human chemokine and cytokine genes using a RT-PCR Array. Fold changes were calculated via ΔΔCt against the control group at day 5 relative to housekeeping genes. At least six lung organoids were analyzed for each group. (**E**) Volcano plot of differentially expressed genes in B.1.1.7 (red) compared to VIDO-01 (blue) infected lung organoids.

To evaluate the importance of the systemic human immune system in mediating viral clearance we implanted TKO mice with subcutaneous human lung organoids in the absence of a systemic human immune system. The lung-only mice (LoM) were challenged with 1 × 10^5^ TCID_50_ of each viral variant and assessed longitudinally for levels of live virus in lungs harvested at days 2, 5, 7 and 9 (Figure 5A). In the absence of the systemic human immune system, infectious virus persisted at day 9 in 100% (VIDO-01) and 83% (B.1.1.7) of the challenged lung organoids (Figure 5A) compared to only 0% (VIDO-01) and 20% (B.1.1.7) of lung organoids at day 9 in TKO-BLT-L mice that were reconstituted with a systemic human immune system (Figure 4A). When assessed for tissue lesions by histology, LoM infected with VIDO-01 had a similar pathology index in lung organoids as BLT-L mice. In contrast, lung organoids from animals infected with B.1.1.7 had a significantly lower pathology index in LoM than in the BLT-L mice that contained a human immune system (Figure 5B). Lung organoids collected at day 5 from TKO-BLT-L mice infected with either VIDO-01 or B.1.1.7 were assessed by IHC for levels of human plasmacytoid dendritic cells (pDC) (BDCA-2), macrophages (CD68), T cells (CD3) and B cells (CD20) compared to uninfected controls. Scoring of the levels of IHC staining indicated a moderate increase in pDCs in infected lungs that did not reach significance whereas macrophages were significantly increased in both variant infections (Figure 5C). Interestingly, T cell staining was significantly increased in the VIDO-01 infected lungs whereas B cell staining was significantly greater in the B.1.1.7 infected lungs (Figure 5C).

Next, the lung organoids collected at day 5 post-infection were evaluated for levels of 84 different human cytokine and chemokines (Supplementary Table S1). Lung organoids infected with VIDO-01 or B.1.1.7 virus both exhibited a significantly greater difference in the expression of CCL19, CCL7 CXCL10, CXCL11, CXCL13 compared to uninfected controls (Figure 5D). However, each virus demonstrated unique modulation of cytokine and chemokine gene expression compared to uninfected controls with VIDO-01 significantly modulating the expression of an additional 12 genes and B.1.1.7 only an additional five compared to uninfected samples (Figure 5D). When we compared the cytokine and chemokine gene expression between the two viruses this difference became even more pronounced with VIDO-01 inducing significantly greater amounts of CCL13, IL-27, IFNG, IL-6, CCL3, CXCL11 and IL-11 than B.1.1.7, which induced significantly greater amounts of MSTN and IL-24 compared to VIDO-01 (Figure 5E). Overall, these findings suggest that the systemic human immune system infiltrates infected human lung organoids and participates in the clearance of viral infection and that the type of immune response that is mediated is dependent upon the infecting SARS-CoV-2 variant.

## 4. Discussion

To create a humanized mouse model that would support infection with SARS-CoV-2 we modified our original TKO-BLT mouse model by subcutaneous implantation of two pieces of human lung tissue into the back of the mouse. The implanted lung tissue grew to its maximal size over the course of ~12 weeks, the same amount of time that the human immune system takes to become fully reconstituted. Ultrasound analysis demonstrated high density blood flow within the lung organoids suggesting that systemically delivered anti-SARS-CoV-2 therapeutics would reach the lung organoid, which we and others have previously demonstrated [44,45].

The peripheral blood of the TKO-BLT-L mouse was well reconstituted with both innate and adaptive human cells comparable with the original TKO-BLT model. Thus, the presence of fully developed human lung organoids did not affect systemic human immune reconstitution. Additionally, we observed significantly fewer hematopoietic cells in pre-implant lung tissue compared to organoids implanted into BLT-humanized mice suggesting the engrafted human immune system populated the lung after implantation. Frequencies of human immune cell reconstitution in the lung organoids was similar to the majority of cell frequencies in the blood, with the exception of total CD45^+^ leukocytes and CD4^+^ T cells, which were significantly lower. This deviated from the frequencies of CD45^+^ and CD4^+^ T cells in the NSG-BLT-L lung model [34,35]. However, it did recapitulate the frequencies of these cell types in healthy human lung tissue that we acquired and similarly assessed by flow cytometry. Whether this is a difference in cell isolation techniques from lung organoids between labs or a true difference between the two BLT-L models remains to be determined.

Despite their ectopic location, lung organoids differentiated and developed structures typical of human lungs. For example, we observed mucin-coated ciliated epithelium in the bronchioles. Mucin is secreted by goblet cells and participates in the protection of the lung via the clearance of exogenous particles, and its presence suggested goblet cells in the lung organoids were functional. Lung organoids developed alveolar structures despite a lack of ventilation. However, the organoids had more interstitial tissue between air spaces than neonatal and adult lungs. Hematopoietic cell types were also observed in the lung organoids. Interestingly, we observed a particularly dramatic increase in macrophages in the lung organoids of immune reconstituted animals compared to pre-implant lung tissue. Lung tissue contains alveolar macrophages and interstitial macrophages that originate from yolk sac or liver-derived monocytes depending on the stage of fetal lung development [46]. In our model, the tissue was at canalicular stage (19–22 weeks) when implanted onto the mice, so it was very likely that the macrophages in the lung organoids mainly differentiated from circulating monocytes originating from the engrafted human immune system. We also observed 2.81% (± 2.91) of monocytes in lung organoids compared to 0.76% (± 0.4) in the blood, suggesting a preferential accumulation of circulating monocytes in the human lung tissue. It has been reported that CD68 (KP-1) not only stains alveolar macrophages, but also neutrophils [47]. However, morphological examination of cell types in H&E-stained lung organoids revealed the presence of few neutrophils, which increased only slightly in the presence of SARS-CoV-2 infection.

SARS-CoV-2 spike protein binds to cell surface ACE2 followed by S1/S2 polybasic cleavage by TMPRSS2 resulting in cellular entry and virus replication. It was suggested that children have lower susceptibility to SARS-CoV-2 infection due to lower expression of ACE2 in their lungs [48]. However, despite the fetal origin of the implanted tissue we detected comparable levels of ACE2 and TMPRSS2 in the lung organoids post-implant compared to an adult human lung sample. Consequently, there was no difficulty in achieving adequate levels of infection due to low expression of essential SARS-CoV-2 entry molecules.

Infected TKO-BLT-L mice showed no clinical signs of disease and cleared the infection within a 10 day period which was akin to many human infections [49] and some animal models [16]. However, the lack of clinical disease may also suggest the infection was isolated to the human lung organoids and did not infect the animals systemically, particularly the murine respiratory system, where it may have provoked disease symptoms. The lack of symptoms is surprising for infection with B.1.1.7 as the N501Y mutation in its spike protein has been shown to extend the SARS-CoV-2 host range primarily to the respiratory tract of wildtype mice [50] and suggests the virus did not spread significantly beyond the human lung organoid. Nevertheless, TKO-BLT-L mice demonstrated many other characteristics of human SARS-CoV-2 infection. For example, it has been reported in humans that the B.1.1.7 variant exhibited longer persistence and higher viral RNA loads than early SARS-CoV-2 lineages as was also observed in our model [51]. The mutations identified in spike protein including D614G and N501Y as well as non-spike regions such as orf6 and orf9b might also contribute to the greater infectivity of the B.1.1.7 variant [3,14]. However, whether direct intra-organoid injection of the N501Y bearing B.1.1.7 variant resulted in sufficient systemic infection to potentially contribute to the greater infectious viral load in the human lung organoid was not evaluated. The TKO-BLT-L model would likely also recapitulate the greater infectivity of the Delta (B.1.617.2) variant and may possibly also exhibit greater lung pathology but this remains to be tested. The Omicron variant (B.1.1.529) has been reported to preferentially infect cells of the upper respiratory tract and to replicate to a lower degree in the lungs contributing to less severe disease [52]. While TKO-BLT-L mice would likely recapitulate the reduced replication in lung tissue and reduced lung pathology, it would not have detected a greater tropism for upper respiratory tract cells by infecting directly into lung organoids. In addition, altered Delta-associated mortality [53,54] is unlikely to be observed in our model due to a lack of systemic disease.

Histological stains showed lymphocyte infiltration in both VIDO-01 and B.1.1.7 samples. Our findings were consistent with findings that SARS-CoV-2 infection caused mononuclear cell infiltration in human and non-human primates as well as other animals models including hamster and ACE2 transgenic mice [18,55,56]. Pathological changes like hyaline membrane formation, type-2 pneumocyte hyperplasia, interstitial fibroblastic proliferation and alveolar lumen edema have been reported in human and non-human primates, while in hamster and ACE2 mice, type-2 pneumocyte hyperplasia, hyaline membrane formation and alveolar wall thickening was reported [18,55,56,57]. We observed immune infiltration, type-2 pneumocyte hyperplasia, desquamated epithelial cells, extravasated erythrocytes and the presence of apoptotic bodies in the interstitia and airways of both VIDO-01 and B.1.1.7 infected lung organoids. However, the early day 5 time point precluded detection of hyaline membrane formation or lung consolidation typical of the acute respiratory distress syndrome associated with COVID-19. In agreement with reports in hamsters [58] we did not see any significant increase in lung pathology induced by the B.1.1.7 variant compared to the early B lineage VIDO-01 virus.

SARS-CoV-2 virus mainly targets ciliated epithelial cells and alveolar type 2 cells that co-express ACE2 and TMPRSS2 in humans [59]. Evidence from additional studies showed that bronchiolar epithelial cells, pneumocytes, alveolar epithelial cells and macrophages can also be infected by SARS-CoV-2 [18,57,60]. In our TKO-BLT-L mouse model, we observed SARS-CoV-2 N protein in epithelial cells and in alveolar macrophages. Macrophages are early producers of anti-viral type I interferons. However, reports show that the production of IFNs in macrophages may be restricted by SARS-CoV-2 [61]. In addition, the CD68 staining did not differentiate between macrophages and neutrophils. Considering the rapid drop in viral load after day 5 and increased frequency of CD68^+^ cells in the lung organoids of infected mice, we hypothesize that infiltrating human macrophages and to some degree neutrophils may play a key role in the rapid clearance of infection in our model. That the viral load was not rapidly suppressed in the mice that were implanted with human lung but not a systemic human immune system strongly suggested viral control was human immune cell mediated and not simply an intrinsic local tissue response.

Distinct immune responses were induced during VIDO-01 and B.1.1.7 infection, with more inflammatory Th1-related cytokines upregulated in VIDO-01 infection that coincided with a significant influx of T cells into the T cell organoid. We observed increased expression of CCL13, CCL3, CXCL11 and CXCL10 which were associated with severe COVID-19 outcome in humans [62]. We also observed increases in CXCL10, CXCL11 and CCL5 which were reported in hamster models [16]. It was interesting that cytokine production was somewhat attenuated in the B.1.1.7 variant compared to VIDO-01, which was also observed in a primate study [63]. In addition, IL-24, a Th2-associated cytokine was significantly upregulated in B.1.1.7 compared to VIDO-01 that may coincide with the significant influx of B cells into the lung organoid during B.1.1.7 infection.

Whether the observed differences in cytokine and chemokine expression were merely transient at day 5 or were consistent features of longitudinal infection requires further evaluation. In addition, although the BLT method of humanization has been shown to produce some of the most functional adaptive immune responses in humanized mouse models (albeit comparatively weak by human standards) during prolonged infection models [30,35,64] it remains to be assessed whether antigen specific B and T cells responses would develop to the relatively short-term SARS-CoV-2 infection and whether these responses also differ between VOCs. It would also be of interest to study NK cell responses in more detail as they have been implicated in mediating protective responses to SARS-CoV-2 [65]. However, one well known caveat of humanized mouse models is the lack of a well-developed human NK cell compartment in the absence of supplemented human IL-15 [66,67]. Additionally, while the cytokine and chemokine responses observed in our model are similar to those reported in other humanized mouse models [37,44,68] and many cytokines and chemokines associated with COVID-19 in humans were upregulated including IL1RN, CCL7, IFN-γ, IFNα2 and CXCL9-11 [69,70], others were absent such as IL-6 and TNFα. Whether this reflects differences in the infecting viruses, sample type (lung tissue compared to bronchial alveolar lavage fluid or blood plasma) or a deficiency of the humanized mouse system remains to be determined. Chemokine and cytokine responses have been reported to be distinct in children and adult COVID-19 [71]. Serum IL-6 was elevated in adult patients with COVID-19 and its level was associated with severity and prognosis [72]. Conversely, reduced inflammatory damage was found in pediatric patients, for example, IL-6 was increased in children with mild symptoms [73]. Thus, the fetal origin of lung organoids may also have affected the immune responses in our model. With the recent global Omicron surge, there have been reports of more pediatric COVID-19 hospital cases. Our model might be particularly useful for the study of SARS-CoV-2 and evaluation of immune responses and therapeutics in relation to pediatric disease.

Other humanized lung mice have been generated on immunocompromised mouse backgrounds for the study of SARS-CoV-2 [37,44,68,74]. To our knowledge, ours is the first bone marrow, liver, thymus (BLT) model to be implanted with lung and used to assess SARS-CoV-2 infection whereas previous SARS-CoV-2 investigations were performed either in models solely implanted with human lung [37,44] and/or were additionally injected with fetal liver CD34^+^ cells to generate human immune system (HIS) mice [68,74]. Direct comparisons between the lung lesions and immune responses are difficult between the models as each type of humanization results in different levels of human immune reconstitution and immune functionality. Additionally, different viral variants and doses were used and different time points often assessed in the limited studies currently available. There is some disparity between the models regarding the contribution of the implanted human immune system to virus-related lesions in the human organoid [44,68,74]. In our model, pathology appeared dependent on the infecting viral variant with B.1.1.7 producing significantly fewer lesions in animals solely implanted with human lung and not a human immune system, whereas VIDO-01 pathology was similar in both situations. Overall and similar to our findings, most models showed generation of pathogenic lesions and induction of cytokines responses that are associated with SARS-CoV-2 infection in humans and a role for the implanted human immune system in clearing the infection [37,44,68,74]. However, it appears imperative to carefully design studies of SARS-CoV-2 and interpret data with the limitations of each specific type of humanized lung mouse model and the specific viral variant being used in mind.

The addition of implanted human lung organoids to our TKO-BLT human immune system mouse model facilitated the comparison of a SARS-CoV-2 variant to an early lineage virus, potentially replicating some aspects with greater fidelity to genuine human disease than is possible in other animal models. However, the animals are not infected though the natural nasal route thereby omitting evaluation of the immune pathobiology of the upper respiratory tract and the apparent isolation of the infection to the human organoid, as indicated by a lack of observable disease symptoms, prevented the evaluation of systemic effects of SARS-CoV-2 infection. In addition to SARS-CoV-2, the TKO-BLT-L mouse model is likely to support other respiratory viral infections such as HCMV, SARS-CoV, MERS, and RSV and to recapitulate pathological changes and immune responses [35,75] specific to human disease in the lung. Overall, the TKO-BLT-L model contained a highly reconstituted human immune system, supported SARS-CoV-2 infection, recapitulated characteristic differences between an early B lineage virus and VOC and revealed previous undescribed differences in immune responses to each variant. Thus TKO-BLT-L model provides a platform to study the SARS-CoV-2 immunopathobiology of VOCs in the context of genuine human lung tissue and immune cells. 

## Figures and Tables

**Table 1:** Comparison of human immune cell reconstitution in the peripheral blood of TKO-BLT and TKO-BLT-L mice at 12 weeks post-surgery.

	Per mL blood	% of Live	% of CD45^+^	% of T Cells		% of CD45^+^					% of DC	
	^2^ CD45	CD45	T Cells	CD4	CD8	CD14	CD33	B Cells	NK cells	DC	mDC	pDC
BLT-L 12 ^1^wps	^3^ 4.69 × 10^5^ (±5.23 × 10^4^)	61.37 (±11.81)	70.84 (±13.91)	73.95 (±4.33)	23.51 (±3.77)	0.76 (±0.40)	4.54 (±2.94)	26.33 (±19.32)	0.27 (±0.24)	3.69 (±2.16)	0.28 (±0.09)	0.29 (±0.18)
BLT 12 wps	4.59 × 10^5^ (±3.08 × 10^5^	58.75 (±14.49)	74.84 (±17.21)	71.13 (±8.72)	16.54 (±12.40)	1.16 (±1.21)	2.93 (±2.38)	19.37 (±15.07)	0.49 (±0.73)	3.27 (±2.32)	0.25 (±0.27)	0.08 (±0.11)
*p*-Value	0.5720	0.7930	0.8971	0.1331	0.5844	0.4738	0.5326	0.7496	0.3589	0.7462	0.5999	0.1248

^1^ weeks post-surgery (wps). ^2^ Data were collected from mouse cohorts produced from four different human donors (n=78 for BLT-L, n=72 for BLT). ^3^ Mean, (±SD). NK: natural killer cells; DC: dendritic cells; mDC: myeloid dendritic cells; pDC: plasmacytoid dendritic cells.

**Table 2:** Human immune cell reconstitution in the lung organoids of TKO-BLT-L mice and an adult human lung.

	% of Live	% of CD45^+^	% of T Cells		% of CD45^+^					% of DC	
	CD45	T Cells	CD4	CD8	CD14	CD33	B Cells	NK	DC	mDC	pDC
^1^ Lung organoid	^3^ 26.31 (±15.42)	64.69 (±13.53)	0.14 (±0.11)	31.50 (±6.38)	2.82 (±2.91)	8.53 (±4.96)	20.80 (±8.37)	0.54 (±0.39)	3.38 (±1.48)	21.83 (±6.94)	0.29 (±0.38)
^2^ Adult lung	11	48	0.46	25	17.1	41.3	3.03	3.04	13.3	2.37	0.87

^1^ Data were collected from mouse cohorts produced from 2 different human donors (n = 11). ^2^ 59-year-old female. ^3^ Mean ± SD. NK: natural killer cells; DC: dendritic cells; mDC: myeloid dendritic cells; pDC: plasmacytoid dendritic cells.

## Data Availability

Not applicable.

## Supplementary Materials

The following supporting information can be downloaded at: https://www.mdpi.com/article/10.3390/v14102272/s1, Table S1: Supplementary Table 1. Change in expression of 84 cytokine and chemokine genes from lung tissues infected with VIDO-01 or B.1.1.7 compared to uninfected tissues.

## References

[1] Thakur V., Ratho R.K. (2021). OMICRON (B.1.1.529): A new SARS-CoV-2 variant of concern mounting worldwide fear. J. Med. Virol..

[2] Harvey W.T., Carabelli A.M., Jackson B., Gupta R.K., Thomson E.C., Harrison E.M., Ludden C., Reeve R., Rambaut A., COVID-19 Genomics UK (COG-UK) Consortium (2021). SARS-CoV-2 variants, spike mutations and immune escape. Nat. Rev. Microbiol..

[3] Zhang L., Jackson C.B., Mou H., Ojha A., Peng H., Quinlan B.D., Rangarajan E.S., Pan A., Vanderheiden A., Suthar M.S. (2020). SARS-CoV-2 spike-protein D614G mutation increases virion spike density and infectivity. Nat. Commun..

[4] Ali F., Kasry A., Amin M. (2021). The new SARS-CoV-2 strain shows a stronger binding affinity to ACE2 due to N501Y mutant. Med. Drug Discov..

[5] Zhou B., Thao T.T.N., Hoffmann D., Taddeo A., Ebert N., Labroussaa F., Pohlmann A., King J., Steiner S., Kelly J.N. (2021). SARS-CoV-2 spike D614G change enhances replication and transmission. Nature.

[6] Thomson E.C., Rosen L.E., Shepherd J.G., Spreafico R., da Silva Filipe A., Wojcechowskyj J.A., Davis C., Piccoli L., Pascall D.J., Dillen J. (2021). Circulating SARS-CoV-2 spike N439K variants maintain fitness while evading antibody-mediated immunity. Cell.

[7] Mok B.W., Liu H., Deng S., Liu J., Zhang A.J., Lau S.Y., Liu S., Tam R.C., Cremin C.J., Ng T.T. (2021). Low dose inocula of SARS-CoV-2 Alpha variant transmits more efficiently than earlier variants in hamsters. Commun. Biol..

[8] Greaney A.J., Loes A.N., Crawford K.H., Starr T.N., Malone K.D., Chu H.Y., Bloom J.D. (2021). Comprehensive mapping of mutations in the SARS-CoV-2 receptor-binding domain that affect recognition by polyclonal human plasma antibodies. Cell Host Microbe.

[9] Liu C., Ginn H.M., Dejnirattisai W., Supasa P., Wang B., Tuekprakhon A., Nutalai R., Zhou D., Mentzer A.J., Zhao Y. (2021). Reduced neutralization of SARS-CoV-2 B.1.617 by vaccine and convalescent serum. Cell.

[10] Garcia-Beltran W.F., Lam E.C., St Denis K., Nitido A.D., Garcia Z.H., Hauser B.M., Feldman J., Pavlovic M.N., Gregory D.J., Poznansky M.C. (2021). Multiple SARS-CoV-2 variants escape neutralization by vaccine-induced humoral immunity. Cell.

[11] Abu-Raddad L.J., Chemaitelly H., Butt A.A., National Study Group for C-V (2021). Effectiveness of the BNT162b2 Covid-19 Vaccine against the B.1.1.7 and B.1.351 Variants. N. Engl. J. Med..

[12] Collie S., Champion J., Moultrie H., Bekker L.-G., Gray G. (2021). Effectiveness of BNT162b2 vaccine against omicron variant in South Africa. N. Engl. J. Med..

[13] Accorsi E.K., Britton A., Fleming-Dutra K.E., Smith Z.R., Shang N., Derado G., Miller J., Schrag S.J., Verani J.R. (2022). Association Between 3 Doses of mRNA COVID-19 Vaccine and Symptomatic Infection Caused by the SARS-CoV-2 Omicron and Delta Variants. JAMA.

[14] Thorne L.G., Bouhaddou M., Reuschl A.K., Zuliani-Alvarez L., Polacco B., Pelin A., Batra J., Whelan M.V.X., Hosmillo M., Fossati A. (2021). Evolution of enhanced innate immune evasion by SARS-CoV-2. Nature.

[15] Munoz-Fontela C., Dowling W.E., Funnell S.G.P., Gsell P.S., Riveros-Balta A.X., Albrecht R.A., Andersen H., Baric R.S., Carroll M.W., Cavaleri M. (2020). Animal models for COVID-19. Nature.

[16] Shou S., Liu M., Yang Y., Kang N., Song Y., Tan D., Liu N., Wang F., Liu J., Xie Y. (2021). Animal Models for COVID-19: Hamsters, Mouse, Ferret, Mink, Tree Shrew, and Non-human Primates. Front. Microbiol..

[17] Pandey K., Acharya A., Mohan M., Ng C.L., Reid S.P., Byrareddy S.N. (2020). Animal models for SARS-CoV-2 research: A comprehensive literature review. Transbound. Emerg. Dis..

[18] Sia S.F., Yan L.M., Chin A.W.H., Fung K., Choy K.T., Wong A.Y.L., Kaewpreedee P., Perera R., Poon L.L.M., Nicholls J.M. (2020). Pathogenesis and transmission of SARS-CoV-2 in golden hamsters. Nature.

[19] Zhao X., Chen D., Szabla R., Zheng M., Li G., Du P., Zheng S., Li X., Song C., Li R. (2020). Broad and differential animal angiotensin-converting enzyme 2 receptor usage by SARS-CoV-2. J. Virol..

[20] Li R., Qiao S., Zhang G. (2020). Analysis of angiotensin-converting enzyme 2 (ACE2) from different species sheds some light on cross-species receptor usage of a novel coronavirus 2019-nCoV. J. Infect..

[21] Mestas J., Hughes C.C. (2004). Of mice and not men: Differences between mouse and human immunology. J. Immunol..

[22] Harari D., Abramovich R., Zozulya A., Smith P., Pouly S., Koster M., Hauser H., Schreiber G. (2014). Bridging the species divide: Transgenic mice humanized for type-I interferon response. PLoS ONE.

[23] Hughes A.L. (1995). The evolution of the type I interferon gene family in mammals. J. Mol. Evol..

[24] Bao L., Deng W., Huang B., Gao H., Liu J., Ren L., Wei Q., Yu P., Xu Y., Qi F. (2020). The pathogenicity of SARS-CoV-2 in hACE2 transgenic mice. Nature.

[25] Jiang R.D., Liu M.Q., Chen Y., Shan C., Zhou Y.W., Shen X.R., Li Q., Zhang L., Zhu Y., Si H.R. (2020). Pathogenesis of SARS-CoV-2 in Transgenic Mice Expressing Human Angiotensin-Converting Enzyme 2. Cell.

[26] Maucourant C., Filipovic I., Ponzetta A., Aleman S., Cornillet M., Hertwig L., Strunz B., Lentini A., Reinius B., Brownlie D. (2020). Natural killer cell immunotypes related to COVID-19 disease severity. Sci. Immunol..

[27] Gomez-Cadena A., Spehner L., Kroemer M., Khelil M.B., Bouiller K., Verdeil G., Trabanelli S., Borg C., Loyon R., Jandus C. (2021). Severe COVID-19 patients exhibit an ILC2 NKG2D+ population in their impaired ILC compartment. Cell. Mol. Immunol..

[28] Allen T.M., Brehm M.A., Bridges S., Ferguson S., Kumar P., Mirochnitchenko O., Palucka K., Pelanda R., Sanders-Beer B., Shultz L.D. (2019). Humanized immune system mouse models: Progress, challenges and opportunities. Nat. Immunol..

[29] Garcia J.V. (2016). Humanized mice for HIV and AIDS research. Curr. Opin. Virol..

[30] Lavender K.J., Pang W.W., Messer R.J., Duley A.K., Race B., Phillips K., Scott D., Peterson K.E., Chan C.K., Dittmer U. (2013). BLT-humanized C57BL/6 Rag2−/− γ c−/− CD47−/− mice are resistant to GVHD and develop B-and T-cell immunity to HIV infection. Blood.

[31] Lavender K.J., Messer R.J., Race B., Hasenkrug K.J. (2014). Production of bone marrow, liver, thymus (BLT) humanized mice on the C57BL/6 Rag2(-/-)gammac(-/-)CD47(-/-) background. J. Immunol. Methods.

[32] Lavender K.J., Pace C., Sutter K., Messer R.J., Pouncey D.L., Cummins N.W., Natesampillai S., Zheng J., Goldsmith J., Widera M. (2018). An advanced BLT-humanized mouse model for extended HIV-1 cure studies. AIDS.

[33] Lavender K.J., Gibbert K., Peterson K.E., Van Dis E., Francois S., Woods T., Messer R.J., Gawanbacht A., Muller J.A., Munch J. (2016). Interferon Alpha Subtype-Specific Suppression of HIV-1 Infection In Vivo. J. Virol..

[34] Wang Y., Wang L., Fu C., Wang X., Zuo S., Shu C., Shan Y., He J., Zhou Q., Li W. (2022). Exploration of Human Lung-Resident Immunity and Response to Respiratory Viral Immunization in a Humanized Mouse Model. J. Immunol..

[35] Wahl A., De C., Abad Fernandez M., Lenarcic E.M., Xu Y., Cockrell A.S., Cleary R.A., Johnson C.E., Schramm N.J., Rank L.M. (2019). Precision mouse models with expanded tropism for human pathogens. Nat. Biotechnol..

[36] Escaffre O., Saito T.B., Juelich T.L., Ikegami T., Smith J.K., Perez D.D., Atkins C., Levine C.B., Huante M.B., Nusbaum R.J. (2017). Contribution of Human Lung Parenchyma and Leukocyte Influx to Oxidative Stress and Immune System-Mediated Pathology following Nipah Virus Infection. J. Virol..

[37] Fu W., Wang W., Yuan L., Lin Y., Huang X., Chen R., Cai M., Liu C., Chen L., Zhou M. (2021). A SCID mouse-human lung xenograft model of SARS-CoV-2 infection. Theranostics.

[38] Ton N., Goncin U., Panahifar A., Chapman D., Wiebe S., Machtaler S. (2021). Developing a Microbubble-Based Contrast Agent for Synchrotron In-Line Phase Contrast Imaging. IEEE Trans. Biomed. Eng..

[39] Sutter K., Lavender K.J., Messer R.J., Widera M., Williams K., Race B., Hasenkrug K.J., Dittmer U. (2019). Concurrent administration of IFNalpha14 and cART in TKO-BLT mice enhances suppression of HIV-1 viremia but does not eliminate the latent reservoir. Sci. Rep..

[40] Van Dis E.S., Moore T.C., Lavender K.J., Messer R.J., Keppler O.T., Verheyen J., Dittmer U., Hasenkrug K.J. (2016). No SEVI-mediated enhancement of rectal HIV-1 transmission of HIV-1 in two humanized mouse cohorts. Virology.

[41] Lavender K.J., Williamson B.N., Saturday G., Martellaro C., Griffin A., Hasenkrug K.J., Feldmann H., Prescott J. (2018). Pathogenicity of Ebola and Marburg Viruses Is Associated With Differential Activation of the Myeloid Compartment in Humanized Triple Knockout-Bone Marrow, Liver, and Thymus Mice. J. Infect. Dis..

[42] Zhou P., Yang X.L., Wang X.G., Hu B., Zhang L., Zhang W., Si H.R., Zhu Y., Li B., Huang C.L. (2020). A pneumonia outbreak associated with a new coronavirus of probable bat origin. Nature.

[43] Hoffmann M., Kleine-Weber H., Schroeder S., Kruger N., Herrler T., Erichsen S., Schiergens T.S., Herrler G., Wu N.H., Nitsche A. (2020). SARS-CoV-2 Cell Entry Depends on ACE2 and TMPRSS2 and Is Blocked by a Clinically Proven Protease Inhibitor. Cell.

[44] Wahl A., Gralinski L.E., Johnson C.E., Yao W., Kovarova M., Dinnon K.H., Liu H., Madden V.J., Krzystek H.M., De C. (2021). SARS-CoV-2 infection is effectively treated and prevented by EIDD-2801. Nature.

[45] Schuhenn J., Meister T.L., Todt D., Bracht T., Schork K., Billaud J.N., Elsner C., Heinen N., Karakoese Z., Haid S. (2022). Differential interferon-alpha subtype induced immune signatures are associated with suppression of SARS-CoV-2 infection. Proc. Natl. Acad. Sci. USA.

[46] Guilliams M., De Kleer I., Henri S., Post S., Vanhoutte L., De Prijck S., Deswarte K., Malissen B., Hammad H., Lambrecht B.N. (2013). Alveolar macrophages develop from fetal monocytes that differentiate into long-lived cells in the first week of life via GM-CSF. J. Exp. Med..

[47] Frafjord A., Skarshaug R., Hammarstrom C., Stankovic B., Dorg L.T., Aamodt H., Woldbaek P.R., Helland A., Brustugun O.T., Oynebraten I. (2020). Antibody combinations for optimized staining of macrophages in human lung tumours. Scand. J. Immunol..

[48] Yonker L.M., Neilan A.M., Bartsch Y., Patel A.B., Regan J., Arya P., Gootkind E., Park G., Hardcastle M., St John A. (2020). Pediatric Severe Acute Respiratory Syndrome Coronavirus 2 (SARS-CoV-2): Clinical Presentation, Infectivity, and Immune Responses. J. Pediatr..

[49] Cevik M., Tate M., Lloyd O., Maraolo A.E., Schafers J., Ho A. (2021). SARS-CoV-2, SARS-CoV, and MERS-CoV viral load dynamics, duration of viral shedding, and infectiousness: A systematic review and meta-analysis. Lancet Microbe..

[50] Shuai H., Chan J.F., Yuen T.T., Yoon C., Hu J.C., Wen L., Hu B., Yang D., Wang Y., Hou Y. (2021). Emerging SARS-CoV-2 variants expand species tropism to murines. EBioMedicine.

[51] Calistri P., Amato L., Puglia I., Cito F., Di Giuseppe A., Danzetta M.L., Morelli D., Di Domenico M., Caporale M., Scialabba S. (2021). Infection sustained by lineage B. 1.1. 7 of SARS-CoV-2 is characterised by longer persistence and higher viral RNA loads in nasopharyngeal swabs. Int. J. Infect. Dis..

[52] Wolter N., Jassat W., Walaza S., Welch R., Moultrie H., Groome M., Amoako D.G., Everatt J., Bhiman J.N., Scheepers C. (2022). Early assessment of the clinical severity of the SARS-CoV-2 omicron variant in South Africa: A data linkage study. Lancet.

[53] Shiehzadegan S., Alaghemand N., Fox M., Venketaraman V. (2021). Analysis of the Delta Variant B.1.617.2 COVID-19. Clin. Pract..

[54] Shuai H., Chan J.F., Hu B., Chai Y., Yuen T.T., Yin F., Huang X., Yoon C., Hu J.C., Liu H. (2022). Attenuated replication and pathogenicity of SARS-CoV-2 B.1.1.529 Omicron. Nature.

[55] Rockx B., Kuiken T., Herfst S., Bestebroer T., Lamers M.M., Oude Munnink B.B., de Meulder D., van Amerongen G., van den Brand J., Okba N.M.A. (2020). Comparative pathogenesis of COVID-19, MERS, and SARS in a nonhuman primate model. Science.

[56] Martines R.B., Ritter J.M., Matkovic E., Gary J., Bollweg B.C., Bullock H., Goldsmith C.S., Silva-Flannery L., Seixas J.N., Reagan-Steiner S. (2020). Pathology and Pathogenesis of SARS-CoV-2 Associated with Fatal Coronavirus Disease, United States. Emerg. Infect. Dis..

[57] Winkler E.S., Bailey A.L., Kafai N.M., Nair S., McCune B.T., Yu J., Fox J.M., Chen R.E., Earnest J.T., Keeler S.P. (2020). SARS-CoV-2 infection of human ACE2-transgenic mice causes severe lung inflammation and impaired function. Nat. Immunol..

[58] Nuñez I.A., Lien C.Z., Selvaraj P., Stauft C.B., Liu S., Starost M.F., Wang T.T. (2021). SARS-CoV-2 B. 1.1. 7 Infection of Syrian Hamster Does Not Cause More Severe Disease, and Naturally Acquired Immunity Confers Protection. Msphere.

[59] Hou Y.J., Okuda K., Edwards C.E., Martinez D.R., Asakura T., Dinnon K.H., Kato T., Lee R.E., Yount B.L., Mascenik T.M. (2020). SARS-CoV-2 Reverse Genetics Reveals a Variable Infection Gradient in the Respiratory Tract. Cell.

[60] Junqueira C., Crespo A., Ranjbar S., de Lacerda L.B., Lewandrowski M., Ingber J., Parry B., Ravid S., Clark S., Schrimpf M.R. (2022). FcgammaR-mediated SARS-CoV-2 infection of monocytes activates inflammation. Nature.

[61] Dalskov L., Møhlenberg M., Thyrsted J., Blay-Cadanet J., Poulsen E.T., Folkersen B.H., Skaarup S.H., Olagnier D., Reinert L., Enghild J.J. (2020). SARS-CoV-2 evades immune detection in alveolar macrophages. EMBO Rep..

[62] Coperchini F., Chiovato L., Ricci G., Croce L., Magri F., Rotondi M. (2021). The cytokine storm in COVID-19: Further advances in our understanding the role of specific chemokines involved. Cytokine Growth Factor Rev..

[63] Rosenke K., Feldmann F., Okumura A., Hansen F., Tang-Huau T.L., Meade-White K., Kaza B., Callison J., Lewis M.C., Smith B.J. (2021). UK B.1.1.7 (Alpha) variant exhibits increased respiratory replication and shedding in nonhuman primates. Emerg. Microbes Infect..

[64] Melkus M.W., Estes J.D., Padgett-Thomas A., Gatlin J., Denton P.W., Othieno F.A., Wege A.K., Haase A.T., Garcia J.V. (2006). Humanized mice mount specific adaptive and innate immune responses to EBV and TSST-1. Nat. Med..

[65] Di Vito C., Calcaterra F., Coianiz N., Terzoli S., Voza A., Mikulak J., Della Bella S., Mavilio D. (2022). Natural Killer Cells in SARS-CoV-2 Infection: Pathophysiology and Therapeutic Implications. Front. Immunol..

[66] Huntington N.D., Legrand N., Alves N.L., Jaron B., Weijer K., Plet A., Corcuff E., Mortier E., Jacques Y., Spits H. (2009). IL-15 trans-presentation promotes human NK cell development and differentiation in vivo. J. Exp. Med..

[67] Chen Q., Khoury M., Chen J. (2009). Expression of human cytokines dramatically improves reconstitution of specific human-blood lineage cells in humanized mice. Proc. Natl. Acad. Sci. USA.

[68] Sun R., Zhao Z., Fu C., Wang Y., Guo Z., Zhang C., Liu L., Zhang C., Shu C., He J. (2022). Humanized mice for investigating SARS-CoV-2 lung infection and associated human immune responses. Eur. J. Immunol..

[69] Callahan V., Hawks S., Crawford M.A., Lehman C.W., Morrison H.A., Ivester H.M., Akhrymuk I., Boghdeh N., Flor R., Finkielstein C.V. (2021). The Pro-Inflammatory Chemokines CXCL9, CXCL10 and CXCL11 Are Upregulated Following SARS-CoV-2 Infection in an AKT-Dependent Manner. Viruses.

[70] Liao M., Liu Y., Yuan J., Wen Y., Xu G., Zhao J., Cheng L., Li J., Wang X., Wang F. (2020). Single-cell landscape of bronchoalveolar immune cells in patients with COVID-19. Nat. Med..

[71] Ozsurekci Y., Aykac K., Er A.G., Halacli B., Arasli M., Oygar P.D., Gurlevik S., Cura Yayla B.C., Karakaya J., Alp A. (2021). Predictive value of cytokine/chemokine responses for the disease severity and management in children and adult cases with COVID-19. J. Med. Virol..

[72] Del Valle D.M., Kim-Schulze S., Huang H.-H., Beckmann N.D., Nirenberg S., Wang B., Lavin Y., Swartz T.H., Madduri D., Stock A. (2020). An inflammatory cytokine signature predicts COVID-19 severity and survival. Nat. Med..

[73] Lu W., Yang L., Li X., Sun M., Zhang A., Qi S., Chen Z., Zhang L., Li J., Xiong H. (2021). Early immune responses and prognostic factors in children with COVID-19: A single-center retrospective analysis. BMC Pediatrics.

[74] Kenney D.J., O’Connell A.K., Turcinovic J., Montanaro P., Hekman R.M., Tamura T., Berneshawi A.R., Cafiero T.R., Al Abdullatif S., Blum B. (2022). Humanized mice reveal a macrophage-enriched gene signature defining human lung tissue protection during SARS-CoV-2 infection. Cell Rep..

[75] Sharma A., Wu W., Sung B., Huang J., Tsao T., Li X., Gomi R., Tsuji M., Worgall S. (2016). Respiratory Syncytial Virus (RSV) Pulmonary Infection in Humanized Mice Induces Human Anti-RSV Immune Responses and Pathology. J. Virol..

