# Supplementary material for: SARS-CoV-2 Variant-Specific Infectivity and Immune Profiles Are Detectable in a Humanized Lung Mouse Model
Source: Viruses. 2022 Oct 16;14(10):2272. doi: 10.3390/v14102272 (PMC9612296; doi:10.3390/v14102272)
Supplement: Supplementary file 1 [file viruses-14-02272-s001.zip › viruses-1922596-supplementary.pdf]

Supplementary Table S1. Change in expression of 84 cytokine and chemokine genes from lung tissues infected with VIDO-01 or B.1.1.7 compared to uninfected tissues. N=6

Gene	VIDO-01		B.1.1.7	
**Symbol**	**Fold-change**	**p-Value**	**Fold-change**	**p-Value**
ADIPOQ	-1.22	0.171568	1.01	0.907886
BMP2	1.16	0.465079	1.02	0.993562
BMP4	1.03	0.828522	1.03	0.711055
BMP6	-2.43	0.034802	-1.5	0.131742
BMP7	-1.54	0.040338	1	0.989621
C5	-1.01	0.878866	-1.32	0.290608
CCL1	1.12	0.56013	-1.72	0.124694
CCL11	1.45	0.600397	-1.25	0.226212
CCL13	2.1	0.011406	-1.74	0.06584
CCL17	-1.8	0.026631	1.05	0.60592
CCL18	1.1	0.343477	-1.09	0.744088
CCL19	2.64	0.015105	2.42	0.048017
CCL2	1.6	0.042325	1.05	0.910992
CCL20	1.62	0.24924	1.34	0.475194
CCL21	1.81	0.042976	1.5	0.053712
CCL22	-1.33	0.670717	-1.17	0.404938
CCL24	-1.34	0.20731	-1.49	0.070233
CCL3	1.68	0.604471	-1.27	0.327054
CCL5	1.57	0.264056	1.45	0.350855

CCL7	3.53	0.166965	3.29	0.162341
CCL8	1.57	0.201765	1.22	0.477165
CD40LG	1.95	0.065598	1.83	0.142195
CNTF	-1.19	0.687103	1.32	0.371653
CSF1	1.34	0.007654	1.19	0.092867
CSF2	1.21	0.298746	-1.01	0.929496
CSF3	-1.38	0.638302	-1.05	0.559213
CX3CL1	1.64	0.021695	-1.17	0.219898
CXCL1	1.47	0.233137	1.08	0.657585
CXCL10	6.05	0.009065	2.58	0.243303
CXCL11	12.46	0.00048	3.88	0.130667
CXCL12	-1.26	0.186215	1.04	0.80504
CXCL13	2.82	0.036062	2.51	0.302466
CXCL16	1.28	0.148373	-1.03	0.920214
CXCL2	1.04	0.972029	-1.11	0.667616
CXCL5	2.83	0.151075	1.06	0.665149
CXCL9	2.8	0.185932	1.56	0.836497
FASLG	2.24	0.010579	1.34	0.529215
GPI	1.05	0.479006	-1.03	0.602029
IFNA2	1.38	0.395046	4.16	0.09247
IFNG	2.65	0.123728	1.3	0.73294
IL10	1.28	0.841445	1.36	0.882182
IL11	1.31	0.287004	-2.33	0.029011

IL12A	-1.25	0.300338	-1.17	0.241885
IL12B	2.17	0.189194	1.19	0.988514
IL13	-1.39	0.261121	1.25	0.512339
IL15	1.3	0.668932	1.14	0.9845
IL16	-1.22	0.362656	-1.01	0.355989
IL17A	-1.68	0.168196	-1.37	0.17517
IL17F	-1.86	0.069491	-1.45	0.054238
IL18	1.19	0.480659	-1.1	0.472168
IL1A	1.13	0.506837	1.09	0.765502
IL1B	1.68	0.114005	1.07	0.729998
IL1RN	2.11	0.076866	1.44	0.406342
IL2	-1.09	0.589472	1.02	0.909815
IL21	1.47	0.766414	1.83	0.225003
IL22	-1.25	0.492843	1.35	0.731609
IL23A	1.12	0.452905	1.49	0.025909
IL24	-1.22	0.541561	2.59	0.001461
IL27	2.79	0.099737	1.21	0.643586
IL3	1.27	0.423416	2.01	0.043093
IL4	1.25	0.641989	1.42	0.6282
IL5	-1.17	0.936844	1.85	0.124499
IL6	1.78	0.286208	-1.2	0.3081
IL7	1.54	0.140579	1.63	0.064962
CXCL8	1.49	0.246146	-1.12	0.894457

IL9	-1.1	0.660985	-1.13	0.23102
LIF	1.48	0.083845	1.81	0.075533
LTA	1.44	0.546769	1.74	0.207815
LTB	-1.22	0.273527	1.23	0.785173
MIF	-1.47	0.027382	-3.11	0.056449
MSTN	-2.41	0.056283	1.21	0.500883
NODAL	-2.08	0.580751	1.24	0.476842
OSM	1.78	0.007042	1.61	0.024129
PPBP	-3.42	0.591546	-1.31	0.868689
SPP1	-1.04	0.860132	-1.2	0.398034
TGFB2	-1.77	0.032973	-1.08	0.73939
THPO	-1.05	0.970532	1.02	0.941281
TNF	1.34	0.382109	-1.34	0.244865
TNFRSF11B	-1.05	0.942791	-1.21	0.722239
TNFSF10	1.55	0.032871	1.18	0.454154
TNFSF11	1.08	0.876478	1.33	0.468666
TNFSF13B	1.6	0.013015	1.42	0.126161
VEGFA	-1.47	0.316325	-1.18	0.455823
XCL1	1.17	0.85442	1.98	0.185543
